# Genetic profiling and PVY resistance identification of potato germplasm resources

**DOI:** 10.3389/fpls.2024.1444281

**Published:** 2024-09-20

**Authors:** Yan Gao, Chenxi Tian, Yizhi Du, Yong Zhao, Rui Jiang, Kai Zhang, Dianqiu Lv

**Affiliations:** ^1^ College of Agronomy and Biotechnology, Southwest University, Chongqing, China; ^2^ Key Laboratory of Germplasm Innovation of Upper Yangtze River, Ministry of Agriculture and Rural Affairs, Chongqing, China; ^3^ Engineering Research Center of South Upland Agriculture, Ministry of Education, Chongqing, China; ^4^ Chongqing Key Laboratory of Biology and Genetic Breeding for Tuber and Root Crops, Chongqing, China

**Keywords:** SSR, genetic cluster analysis, PVY, resistance, potato

## Abstract

Excellent germplasm resources are the foundation for cultivating high-quality, disease-resistant, and stress-tolerant varieties. In this study, simple sequence repeat (SSR) markers were used to identify 138 potato accessions collected from worldwide, and genetic cluster analysis was used to characterize the genetic diversity of the tested germplasm resources. The Potato virus Y (PVY) resistance of these potato accessions was identified by artificial friction inoculation combined with molecular marker detection, and potato accessions with different PVY resistance were screened based on disease index and incidence rate. Using SSR markers, 138 potato accessions were identified, and the results showed that the genetic distances between the tested potato germplasm resources ranged from 0.025 to 0.660, and the genetic similarity coefficients ranged from 0.489 to 0.975. The 138 accessions could be clustered into five subgroups using Unweighted Pair-Group Method with Arithmetic Mean (UPGMA). Among them, Z173, Biyin No. 4, Suyin No. 2, XN995, XN987, Biyin No 22, Bibiao104, Sarpo mira, XN996, XN979, Desiree, RUNSHI, Actrice, Jia 1219, Heyin No 12, and Moyin No.1 have relatively distant genetic relationship with another 122 accessions. Based on the disease index, the following different accessions were screened: five highly resistant, 11 resistant, 45 moderately resistant, 35 susceptible, and 42 highly susceptible. Fourteen resource materials with good resistance (disease index ≤ 33.74%, and a grading of high resistance (HR) or medium resistance (MR); incidence rate ≤ 67.58%) were identified. By combining genetic cluster analysis and PVY resistance identification, six accessions showed PVY resistance and had distant genetic relationships with other accessions selected which provided important materials for disease resistance breeding and quality improvement of potato. In this study, the genetic diversity and PVY resistance of global potato germplasm resources was explored, and potato germplasm materials with important utilization value were screened. The results obtained in this study could provide important references for the research and utilization of global potato germplasm resources.

## 1 Introduction

Crop germplasm resources are the genetic resources and material foundation for breeding high-quality, high-yield and high-resistance crop varieties. Collection, protection, accurate identification and evaluation of germplasm resources is essential for fully exploring and utilizing excellent germplasm resources for breeding and scientific research, and promoting seed industry development and technological innovation.

Potato (*Solanum tuberosum* L.) is the fourth largest crop after rice, wheat, and corn (Kleinwechter et al., 2016; Srivastava et al., 2019). Potatoes have a wide range of uses, not only as a main food to meet daily needs, but also playing an important role in various fields such as brewing, medicine, casting, and textiles. In addition, potatoes also benefit the human body as nutrient supplements and antioxidants (Tian et al., 2016). However, potato production is affected by many factors and still needs to be improved. Data collected from 19 regions worldwide between 2001 and 2003 estimated that the potential losses from insect pests, pathogens and viruses in potatoes were 44.9% (Caruana et al., 2021).

Dramatic yield reduction of higher than 50% has been observed in potato due to various diseases especially viruses (Saidi and Hajibarat, 2021), among which, potato virus Y (PVY) is known to be the most harmful disease, causing a significant economic impact on potato crops (Kehoe and Jones, 2016; Kreuze et al., 2020). It can result in significant losses in quality and up to 80% tuber yield loss (Kolychikhina et al., 2021),while the emergence of new strains has accelerated the yield loss in the world (Hosseini et al., 2011). The host range of PVY is very wide, and it can infect at least 163 plant species in 34 genera, with Solanaceae plants being the main ones (Song et al., 2016). PVY is transmitted by over 50 species of aphids, machinery, tools, and by brushing plants while walking through the field (Slater et al., 2020). After being infected with the virus, potato may exhibit symptoms such as potato mosaic, dwarfing, and wrinkling, resulting in significant losses in yield and quality.

At present, there is a lack of effective prevention and control methods for potato virus disease, mainly relying on the resistance of germplasm resources, or producing high quality seed potatoes by virus-free tissue culture. Widespread collection, screening, and creation of potato germplasm resources, as well as precise evaluation and identification of their PVY resistance, are essential for breeding of potato varieties resistant to PVY, and are of great significance for promoting the improvement of potato yield and quality, and promoting seed industry vitalization and potato industrial development.

Molecular markers have been extensively used in genome-wide association study (GWAS) (Zhang et al., 2024), co-dominant markers development (Lin et al., 2021), genetic diversity analysis (Shah et al., 2023), fingerprint construction (Han et al., 2020; Zhao, 2020), genetic linkage maps construction (Li et al., 2019), and molecular breeding (Li et al., 2023), providing favorable theoretical and technical support for plant research and breeding.

Conventional breeding usually involves crossbreeding different varieties or lines, and selecting excellent lines through phenotype observation, resistance identification, and quality measurement of offspring. However, this method not only requires breeders to have rich experience, but also requires complex operations, which lead to low breeding efficiency and long breeding cycles. Molecular marker assisted selection can be used to efficiently screen the target crop germplasm resources with target traits, which can greatly shorten the breeding cycle and improve breeding efficiency. Saidi and Hajibarat (2021) found that the integration of RNA-sequencing (RNA-seq) with GWAS could facilitate the identification of functional single nucleotide polymorphisms (SNPs) which are correlated with rare alleles associated with phenotypes for resistance to viruses and can be utilized for potato breeding and in designing future potato improvement.

There are many resistance genes related to PVY, among which *Ry_adg_* (Munoz et al., 1975), *Ry_sto_* (Grech-Baran et al., 2020), and *Ry_chc_* (Asama et al., 1982) are the main resistance genes. *Ry_adg_* comes from Andean cultivated variety *S. andigena* (Kasai et al., 2000), which is located on chromosome XI (Hämäläinen et al., 1997); *Ry_sto_* comes from *S. stoloniferum*, which is located on chromosome XII (Song et al., 2005; Flis et al., 2005); *Ry_chc_* was discovered from the Japanese variety “Konafubuki”, located at the end of chromosome 9 (Sato et al., 2006). At present, researchers have developed multiple molecular markers for these potato PVY disease resistance genes, to detect whether these resistance genes are present in potato germplasm resources. Among them, markers RYSC3 and RYSC4 used to detect the resistance gene *Ry_adg_* (Kasai et al., 2000; Fulladolsa et al., 2015); YES3-3A, YES3-3B, as well as CAPS markers developed from GP122 and the SSR marker STM0003 used for detecting the resistance gene *Ry_sto_* (Song and Schwarzfischer, 2008; Valkonen et al., 2007); marker Ry186 used to detect extreme resistance gene *Ry_chc_* (Mori et al., 2011).

The application of marker assisted selection (MAS) in PVY resistance breeding was carried out earlier in European countries. The marker RYSC3 developed by Kasai et al. (2000), which is closely linked to the *Ry_adg_* gene, has been widely used in potato breeding abroad. Fulladolsa et al. (2015) evaluated 46 potato lines using markers RYSC3 and YES3-3B, and identified 19 PVY resistant lines. Elison et al. (2020) developed and applied a multi-marker detection method using markers RYSC3, YES3-3A, and Ry186. In China, Bai and Guo (2017) tested 140 potato germplasm resources using the PVY resistance marker RYSC3 and found that 28 of them contained the marker RYSC3. Zhao (2012) used the PVY resistance marker RYSC3 to identify domestic and foreign germplasm resources, and obtained germplasm resources containing the RYSC3 marker, including 22 domestic varieties and 202 imported germplasm resources. Li et al. (2017) used molecular markers RYSC3 and Rxsp, closely linked to *Ry_adg_* and *R_x_*, to conduct marker detection on variety Qingshu 9, Atlantic and their hybrid F_1_ generation. The results showed that the parent Qingshu 9 only contained RYSC3 markers, while the parent Atlantic only contained Rxsp markers. Jiang et al. (2022) used PVY resistance markers RYSC3 and YES3-3A, combined with PVY artificial inoculation identification and screened out 10 potato resources with PVY resistance and high yield suitable for processing potato chips. Lou et al. (2023) used molecular markers closely linked to resistance genes *Ry_adg_*, *Ry_sto_*, *Ry_chc_*, and *R_x_* to conduct marker detection on 102 potato varieties.

Most research on potato germplasm was mainly carried out on germplasm resources for one country or one region. There are few reports on genetic diversity analysis and PVY resistance identification of worldwide potato germplasm resources. In this study, 138 potato germplasm resources were collected from several countries worldwide, and their genetic diversity was assessed based on genetic cluster analysis, and PVY resistance was identified, and germplasm resources with PVY resistance and genetic diversity were screened. The research results can provide important references for the research and utilization of potato germplasm resources, providing germplasm as a base for breeding of disease-resistant and high-quality potato varieties.

## 2 Materials and methods

### 2.1 Plant materials

A total of 138 potato accessions from different countries (**Supplementary Table 1**) were provided by Chongqing Key Laboratory of Biology and Genetic Breeding for Tuber and Root Crops, and the Beibei Resource Bank of potato and sweet potato germplasm resource in Chongqing. The germplasm accessions were collected from 16 countries worldwide, from China (58), the Netherlands (28), Belgium (8), Canada (10), the United States (8), Japan (8), the United Kingdom (8), North Korea (1), Russia (1), Norway (2), Switzerland (1), Ukraine (1), Ireland (1), Morocco (1), South Korea (1), and Denmark (1).

Potato germplasm resources were planted in the Sweet Potato Base H (126.62’N, 45.69’E) in Heilongjiang for PVY resistance identification, and were planted in the Sweet Potato Base X (29.46’N, 106.21’E) in Xiema Town, Beibei District, Chongqing for DNA sample collection.

### 2.2 Genomic DNA extraction and SSR marker detection

Genomic DNA was extracted from fresh young leaves following the CTAB protocol with slight modification (Doyle and Doyle, 1990). SSR marker assay was performed as previously reported (Tang et al., 2016). The PCR reactions were carried out in a total volume of 10 μL containing 5 μL 3G Taq Master Mix, 1 ng DNA template, 0.5 μM of each primer, and ddH_2_O. The PCR amplifications were performed in a 9700 Thermal Cycler (ABI, USA) under the procedure as follows: PCR amplifications pre-denaturation at 94°C for 5 min; 35 cycles of denaturation at 94°C for 45 s, 55°C annealing for 45 s, and extension at 72°C for 1 min; and a final extension at 72°C for 5 min, preservation at 4°C. The SSR primers used in this study shown in **Supplementary Table 2**. PCR products were analyzed on 10% polyacrylamide gel electrophoresis (PAGE) and visualized by silver staining. Band size was estimated using a 100 bp DNA ladder (Tiangen Biotech, Beijing). Polymorphic bands were used to assign loci for each primer and scored as present (1) or absent (0).

### 2.3 Genetic diversity analysis

Genetic similarity coefficient and genetic distance (Nei’s) for 138 potato accessions were calculated using NTsys2. 10 software. Cluster analysis on the potato accessions was done with Unweighted Pair-Group Method with Arithmetic Mean (UPGMA). A cluster analysis tree was constructed and modified using interactive tree of life (ITOL).

### 2.4 PVY resistance identification using artificial inoculation

Plant virus-free potato seedlings that had been tested and confirmed to be virus-free were planted in a 30 cm diameter bowl, when they had five to six leaves, then friction inoculation was applied according to the method of Slater et al. (2020). Potato properties were recorded, PVY incidence rate, disease index and resistance grade were calculated with the following formulae below. The grading of PVY resistance was based on the classes developed by the International Potato Center (CIP), as shown in **Supplementary Table 3**.

Calculation formula:


$$\text{Incidence rate}=\frac{\text{number of diseased plants}}{\text{total number of investigated individuals}}\;\times \;100\%$$

$$\text{Disease index}=\frac{\sum \left(\text{Disease strains at all levels×disease level}\right)}{\underset{\text{multiplied by the highest number of plants}}{\text{total number of surveyed individuals}}}\;\times \;100\%$$

### 2.5 PVY resistance identification using molecular markers

Nine molecular markers linked to potato PVY resistance genes (**Supplementary Table 4**) were selected for PVY resistance gene identification. The PCR reaction was performed using the annealing temperature of each primer pair shown in **Supplementary Table 4**. The PCR reaction system consisted of 5 μL 3G Taq Master Mix, 10 ng DNA template, 0.5 μM of each primer, and ddH_2_O. PCR amplifications were performed in a 9700 Thermal Cycler (ABI, USA) under the following cycle profile: pre-denaturation at 94°C for 5 min; 35 cycles of denaturation at 94°C for 45 s, Tm annealing for 45 s, and extension at 72°C for 1 min; and a final extension at 72°C for 5 min, preservation at 4°C. The amplicons were detected using a 1% agarose gel electrophoresis.

## 3 Results

### 3.1 Genetic diversity analysis of potato germplasm resources

#### 3.1.1 SSR primer polymorphism detection

Forty-one pairs of SSR primers with good reproducibility and high polymorphism were selected and used to amplify the genomic DNA of 138 potato germplasm resources. The amplification fragment size of each marker primer ranged from 100 to 800 bp, and the number of polymorphic loci ranged from 1 to 8. On average, 3.75 polymorphic loci were obtained from each primer pair. The highest number of polymorphic loci were obtained by amplifying the two pairs of primers labeled St-24 and St-42, both of which generated eight polymorphic loci. The above results indicate that these SSR primers have clear amplification bands and good polymorphism, which the detection results can be used for subsequent genetic group analysis of potato germplasm resources.

#### 3.1.2 Genetic distance analysis of potato germplasm resources

According to the SSR marker detection results, the genetic distance and similarity coefficients were calculated using software NTsys2. 10. The genetic similarity coefficients of 138 potato accessions tested ranged from 0.489 to 0.975, and the genetic distances were between 0.025 and 0.659, indicating significant genetic background differences among the potato accessions (**Supplementary Table 5**). Among them, the genetic similarity coefficient between germplasm material XN1021 (No. 102) and Heyin No.17 (No. 103) was the highest, with a value of 0.975; The genetic distance was relatively small, at 0.025, indicating that the genetic relationship between these two materials is closest and the genetic difference is small; The genetic similarity coefficient between resource material Z173 (No. 132) and Burbank No.2 (No. 78) was the smallest, 0.489. The genetic distance was relatively large, 0.660, indicating that these two resource materials have the farthest genetic relationship and significant genetic differences.

#### 3.1.3 Genetic group analysis of potato germplasm

**Figure 1:**
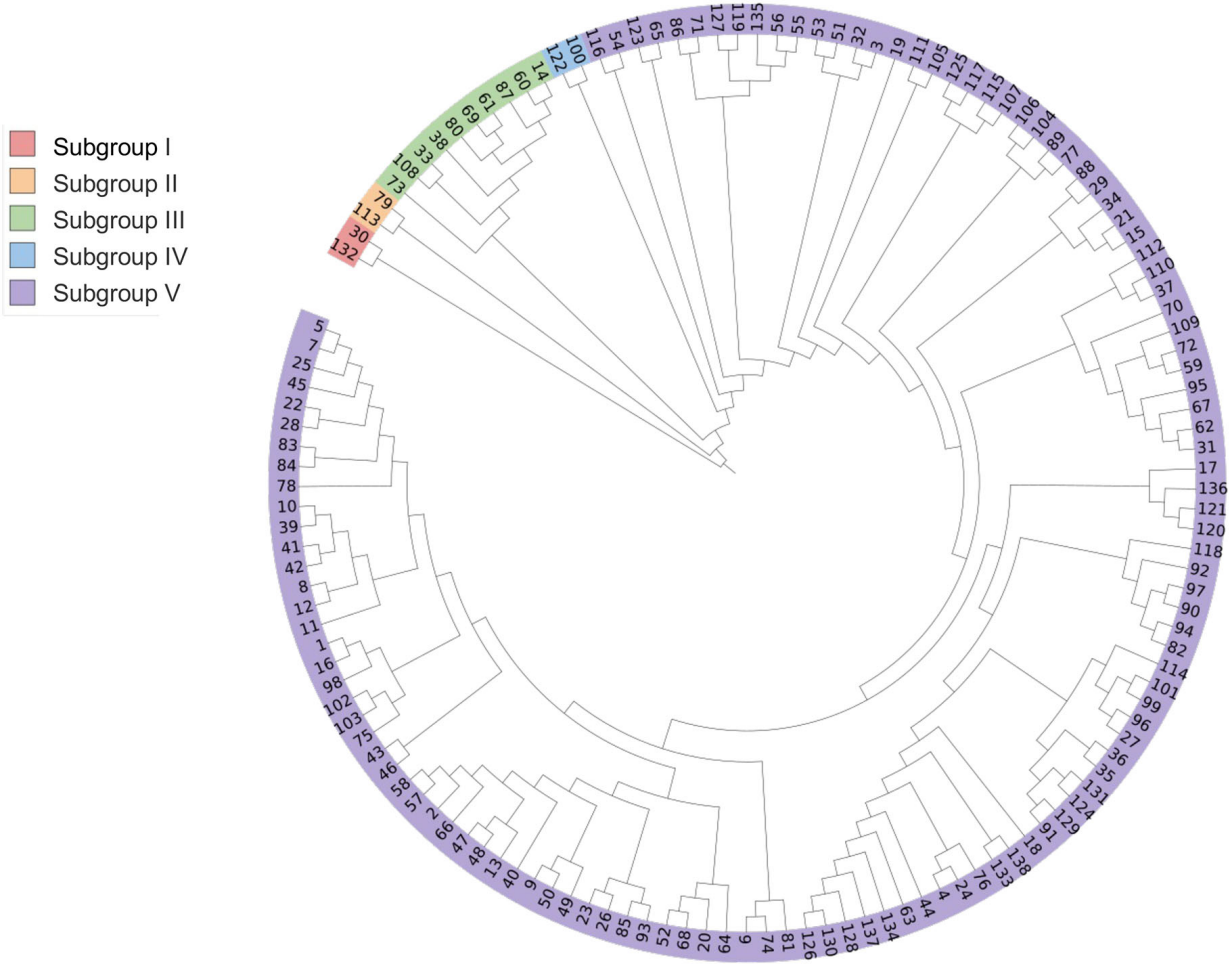
Cluster analysis of 138 potato germplasm accessions.

Based on Nei’s genetic distance, UPGMA method was used to conduct genetic group analysis on 138 potato accessions. As shown in **Figure 1**, the tested potato accessions (their countries of origin is shown in brackets) can be divided into five subgroups. Subgroup I consists of two potato accessions, including Z173 (No. 132, from China) and Biyin No.4 (No. 30, Belgium); Subgroup II is also composed of two potato accessions, including Suyin No.2 (No. 113, the UK) and XN995 (No. 79, China); Subgroup III consists of 10 potato accessions, XN987 (No. 73, China), Biyin No.22 (No. 108, Belgium), Bikang Bionica (No. 33, Belgium), Sarpo mira (No. 38, Denmark), XN996 (No. 80, China), XN979 (No. 69, China), Desire (No. 61, the Netherlands), RUNSHI (No. 87, Switzerland), Actrice (No. 60, the Netherlands), and Jia 1219 (No. 14, Canada); Subgroup IV consists of two potato accessions, Heyin No.12 (No. 100, the Netherlands) and Moyin No.1 (No. 122, Morocco); Subgroup V contains the remaining 122 potato accessions. Analysis of the potato accessions from different subgroups shows that the potato accessions from the same country could be classified into different subgroups, and germplasm from different countries also could be classified into the same subgroup, indicating that potato germplasm from the same country may also have distant genetic relationships or significant genetic background differences, while the potato germplasm from different countries may also have close genetic relationships or similar genetic backgrounds.

### 3.2 Evaluation of PVY resistance of potato germplasm

#### 3.2.1 Identification of resistance to PVY by artificial inoculation of potato germplasm

##### 3.2.1.1 Disease index and incidence rate analysis of potato germplasm

In order to understand the PVY resistance of potato germplasm resources, 138 potato accessions were identified for PVY resistance by artificial inoculation. The results showed that the disease index of 138 potato accessions ranged from 3.82 to 92.76, and the incidence rate of PVY ranged from 32.56 to 99.97%, indicating that there was a large difference in disease index among potato accessions. The disease index of potato germplasm No. 96, 27, 34, 91, and 111 was significantly lower than other germplasm accessions (**Supplementary Table 6**).

From the analysis of resistance index, it can be concluded that among the 138 potato germplasm accessions, there are five highly resistant genotypes (No. 96, 27, 34, 91, and 111), 11 disease resistant accessions (No.9, 20, 24, 59, 130, 104, 103, 114, 118, 132, and 136), 45 accessions of intermediate resistance (No. 117, 35, 71, 101, 97, 2, 62, 38, 124, 64, etc.), 35 susceptible accessions and 42 highly susceptible accessions. It was shown that there were significant differences in incidence rate among different potato accessions. Among them, the incidence rate of potato accessions, No.111, 91, 20, 130, 27, 13, 96, 33, 136, 34, 88, 103, 84, 104 and 133 was significantly lower than of other accessions (**Supplementary Table 7**).

##### 3.2.1.2 Cluster analysis of potato germplasm resources based on PVY resistance

**Figure 2:**
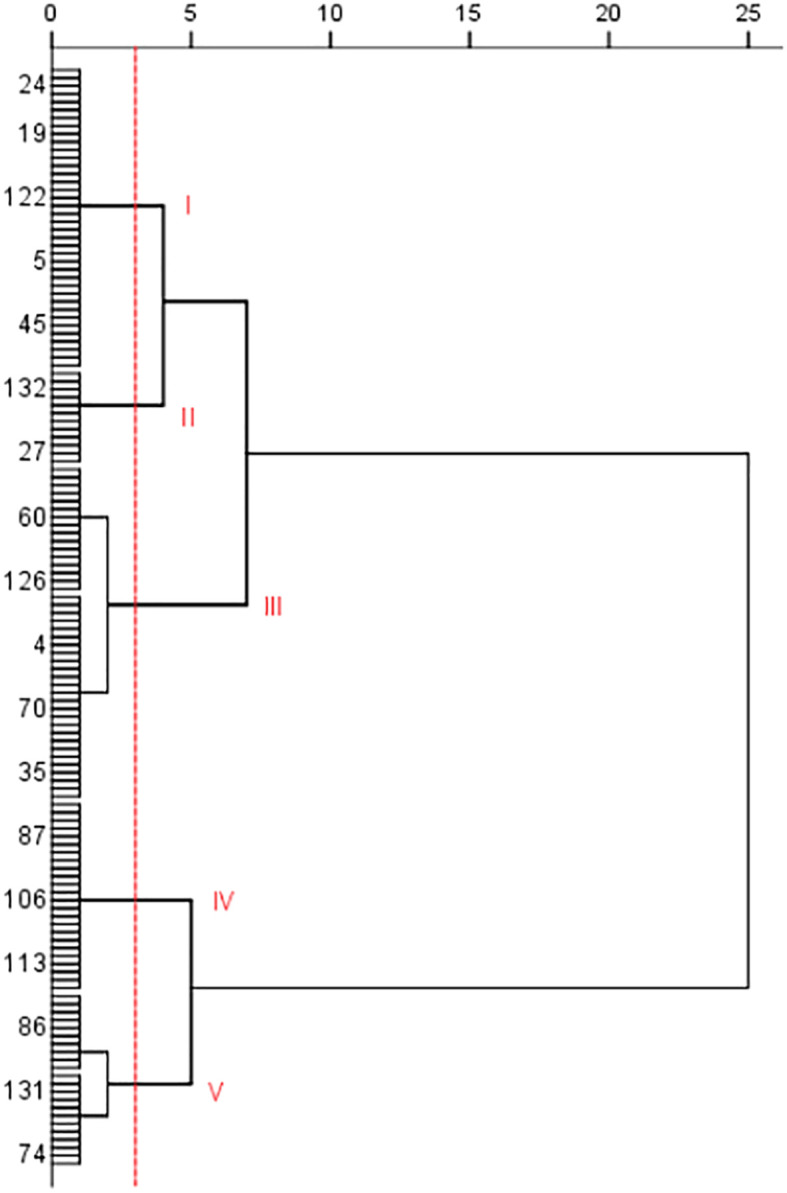
Cluster analysis of tested potato germplasm accessions based on PVY resistance (only some germplasm resource numbers were shown in the figure).

Cluster analysis was conducted on the 138 potato accessions based on the disease index, and the potato accessions were clustered into five categories or types (**Figure 2**). Type I includes 38 potato accessions (such as No. 24, 118, 130, 9, and 13). The disease index range was from 19.94 to 27.07. Type II includes 12 accessions, such as No. 96, 27, 34, 111, and 91, with disease index ranging from 3.82 to 14.24. Type III includes 42 accessions, such as No.116, 64, 124, 38, 2, and 62, with disease index ranges from 32.61 to 53.48. Type IV includes 24 accessions, such as No. 63, 83, 90, 99, 26, and 107, with disease index ranging from 59.75 to 66.84. Type V includes 22 accessions, such as No. 131, 48, 109, 41, 7, 29, and 50, with disease index range from 73.31 to 92.76. The PVY resistance of the five types is in the following order: Type II>Type I>Type III>Type IV>Type V. Type V had the least resistance to PVY.

#### 3.2.2 Molecular marker identification of PVY resistance genes in potato germplasm

**Figure 3:**
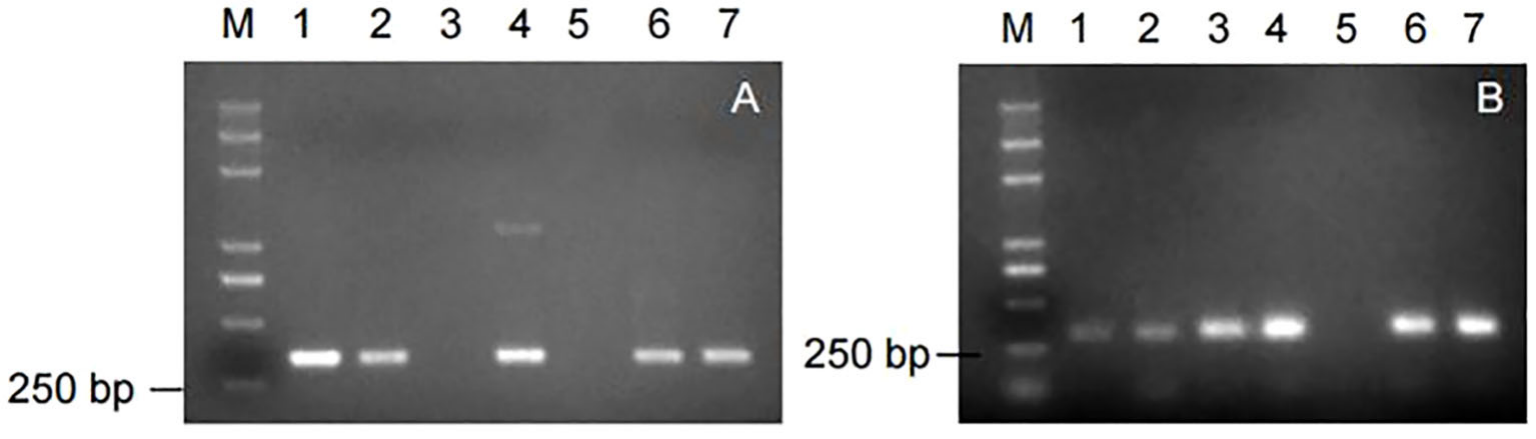
Amplicons of markers in genomic DNA of some potato accessions. **(A)** Amplicons of marker RYSC3; **(B)** Amplicons of marker YES3-3B.

Nine markers were used to amplify the genomic DNA of some potato germplasm materials. Among them, the markers, RYSC3 and YES3-3B, were able to amplify the target band of the expected size, with clear and good repeatability. The amplification results of the other seven marker primers showed poor repeatability or could not amplify the target band. The amplification results of the marker RYSC3 linked to the potato PVY resistance gene *Ry_adg_* are shown in **Figure 3A**. A specific fragment of about 321 bp could be obtained using the genomic DNA of the potato accessions containing the RYSC3 marker as a template. The detection results of the marker YES3-3B linked to the potato PVY resistance gene *Ry_sto_* are shown in **Figure 3B**. A specific fragment of about 284 bp could be obtained using the genomic DNA of the potato accessions containing the YES3-3B marker as a template.

The results showed that in the 138 potato accessions, eight of them contained only RYSC3 markers in their genomic DNA, accounting for 5.80% of the tested germplasm, including accessions No. 5, 27, 29, 49, 76, 101, 132, and 136. There were four accessions containing only YES3-3B markers, accounting for 2.90% of the tested germplasm, including accessions No. 35, 46, 71, and 91. There were 117 accessions, each containing both RYSC3 and YES3-3B markers, accounting for 84.78% of the tested germplasm, including genotypes such as No.1, 2, 3, 6, 7, 8, 9, and 10. There were 10 accessions without any of the two markers, accounting for 6.52% of the tested germplasm, including accessions No. 4, 13, 55, 64, 65, 77, 89, 123, and 131.

#### 3.2.3 Comprehensive analysis of PVY resistance in potato germplasm based on artificial inoculation and molecular markers

The PVY resistance identification of potato germplasm results based on molecular marker analysis showed that 61 potato accessions were identified as resistant (high resistance, disease resistance, medium resistance) by artificial inoculation, of which 56 (91.80%) could detect specific fragments using RYSC3 marker in their genomic DNA, and 54 samples (88.52%) were able to detect specific fragments of marker YES3-3B in their genomic DNA. Seventy-seven potato accessions were identified as susceptible based on their incidence rate, of which eight potato tested negative using marker RYSC3, and 10 tested negative with marker YES3-3B. Other accessions could be classified specific fragments of the two markers (**Table 1**).

**Table 1:** Comparison of PVY resistance identification results of potato germplasm using artificial inoculation and molecular markers.

PVY resistance	Marker	Detection results using molecular markers	Quantity of accessions	Proportion (%)
PVY resistant	RYSC3	+	56	91.80%
		–	5	
	YES3-3B	+	54	88.52%
		–	7	
PVY sensitive	RYSC3	+	69	
		–	8	10.39%
	YES3-3B	+	67	
		–	10	12.99%

The correlation analysis between disease scores and molecular markers is shown in **Table 2**. The phenotype results of PVY resistance in potato accessions are positively correlated with markers RYSC3 and marker YES3- 3B, but not significantly. However, the detection results of marker RYSC3 and marker YES3-3B showed a significant positive correlation (*r* < 0.01).

**Table 2:** Correlation analysis between detection results of RYSC3 and YES3-3B markers and PVY Identification by artificial inoculation.

	Disease index	RYSC3	YES3-3B
Disease index	1	0.057	0.041
RYSC3		1	0.583**
YES-3B			1

**indicates a significant correlation at the 0.01 level.

Based on the results of PVY resistance identification and genetic clustering analysis, it can be concluded that there was no PVY immune (I)-type germplasm identified, indicating that none of the tested potato germplasm was immune to PVY. The distribution of the other accessions with different types of disease resistance also had a certain regularity in the genetic cluster analysis. Specifically, all five PVY HR-type accessions were assigned to subgroup V in the genetic cluster analysis, indicating a close genetic relationship and high similarity in PVY resistance among them. Among the R-type germplasm resources, only germplasm resource No. 132 belongs to group I, while the remaining 11 germplasm resources belong to subgroup V, indicating that No. 132 may have a unique disease resistance mechanism or combination of disease resistance genes. Among the MR-type germplasm, two accessions, No. 79 and No. 113 belong to subgroup II in the genetic group analysis, six accessions, No. 38, 73, 33, 108, 61, and 69 belong to subgroup III, and one accession, No. 122, belongs to subgroup IV, indicating that the germplasm with MR-type showed wide genetic background. Among the germplasm of the S-type, accessions, 341 No. 60 and 80 belong to subgroup III. No. 100 belongs to subgroup IV, and all the other accessions belong to subgroup V. Similarly, among the HS-type germplasm, accession No. 30 belongs to group I, No. 14 and 87 belong to type III, while the remaining accessions belong to subgroup V.

In the genetic cluster analysis, Z173 (No. 132) and Biyin No.4 (No. 30) were divided into subgroup I, Suyin No.2 (No. 113) and XN995 (No. 79) were divided into subgroup II, Sarpo mira (No. 38), Desiree (No. 61) and 10 other accessions were separately divided into subgroup III, and Heyin No.12 (No. 100) and Moyin No.1 (No. 122) into subgroup IV. The average genetic distances between these 16 potato genotypes and the remaining 122 accessions in subgroup V were 0.500, 0.303, 0.298, 0.298, 0.303, 0.342, 0.278, 0.356, 0.271, 0.322, 0.287, 0.250, 0.297, 0.427, 0.265 and 0.289, respectively, which were all higher than the average genetic distance between the tested potato germplasm (0.222). Furthermore, these accessions came from different countries or were bred by different research institutions, which can serve as breeding parents to expand the genetic basis of potato germplasm resources. At the same time, 14 accessions (No. 130, 27, 136, 96, 34, 120, 10, 40, 122, 79, 38, 61, 108, and 132) with good resistance (disease index ≤ 33.74%, and the type of PVY resistance is MR or HR; incidence rate ≤ 67.58%) were screened by combining the disease index and incidence rate.

## 4 Discussion

### 4.1 Genetic cluster analysis of potato germplasm

In this study, 41 SSR molecular markers were used to conduct polymorphism analysis on 138 potato accessions, obtaining a total of 154 polymorphic loci. On average, each SSR molecular marker can generate 3.75 polymorphic loci. Duan (2017) used 36 SSR molecular markers and amplified DNA extracted from 559 potato genotypes as templates, obtaining a total of 134 polymorphic loci. The number of polymorphic loci obtained from each marker ranged from 1 to 7, with an average of 3.72 polymorphic loci per primer pair. The number of polymorphic loci obtained by SSR molecular marker amplification in this study is consistent with previous studies, indicating good polymorphism of these SSR markers.

The genetic diversity of potato can be reflected from multiple characteristic aspects such as morphological, physiological, cytological characteristics, and DNA sequences. Among them, DNA diversity is the essence of genetic diversity, so the application of molecular marker technology is crucial for the analysis of genetic diversity (Liu et al., 2022). SSR marker is a widely used molecular marker, which has the characteristics of low cost, simple operation, good repeatability, and high polymorphism. It is widely used in new variety breeding and genetic diversity research (Zhu, 2013).

By calculating the genetic similarity coefficient and genetic distance, the genetic relationship between potato germplasm resources can be preliminarily determined, which helps to understand the genetic diversity and background of potato germplasm resources. In this study, the genetic distance between potato accessions from 16 countries ranged from 0.025 to 0.660, with an average value of 0.222; The genetic similarity coefficient ranged from 0.489 to 0.975, with an average genetic similarity coefficient of 0.800, indicating that the germplasm accessions tested in the study have relatively rich genetic diversity. Wang Y. et al. (2019) calculated the genetic distance between 292 diverse potato varieties, found their genetic distance ranging from 0.1068 to 0.4558, with an average of 0.309. Yang (2006) calculated the genetic distance between 44 potato varieties, ranging from 0.147 to 0.741. The results of this study indicate that the genetic distance range among 138 potato accessions is relatively large, but the average genetic distance is lower than the average genetic distance between the potato germplasm reported in previous studies. This may be due to a large number of germplasm resources used in this study, but a limited number of SSR molecular markers were used, which did not fully explain the genome-wide differences in the genetic background of the studied germplasm.

Using potato materials with distant genetic distances or significant differences in genetic backgrounds for breeding can effectively expand the genetic diversity of potato (Wang P. et al., 2019). Our results revealed 16 accessions separately divided into subgroup I to IV, and showed distant genetic distance with another 122 tested accessions. These 16 accessions can be used as parents in future breeding research to broaden the genetic diversity of potato germplasm resources in China.

The cluster and genetic diversity analysis of 138 potato accessions in this study helps to deeply understand the genetic information of potato germplasm, providing a reference for parental selection in potato breeding, effectively improving the efficiency of parental selection in potato breeding research, and accelerating the potato breeding process. On the basis of this study, further research for exploring the genetic relationships between potato germplasm at the whole genome level, and evolution analysis using more robust markers such as SNPs, will provide more useful information for efficient potato breeding.

### 4.2 Evaluation of PVY resistance in potato germplasm resources

PVY is one of the most serious viruses harmful to potato production, seriously affecting the yield and quality of potato. The production practices have proven that breeding of varieties with PVY resistance is the most economical and effective method for preventing PVY, and screening germplasm resources resistant to PVY is an important step in PVY-resistance breeding. At present, the screening of PVY-resistant materials mainly relies on field resistance identification and artificial inoculation resistance identification. Compared with the field test, artificial inoculation identification takes less time and is not easily affected by the environment or weather. The conditions of experimental process are easy to control (Mao, 2009). Therefore, we used artificial inoculation to identify the resistance of the 138 accessions. The combination of disease index, incidence rate and molecular markers can more accurately identify PVY resistance and screen PVY-resistant accessions. Based on the disease index and incidence rate, 14 germplasm accessions with good resistance (disease index ≤ 33.74%, and the type of PVY resistance is MR or HR; incidence rate ≤ 67.58%) were screened, and provided good plant materials for PVY-resistance breeding.

Molecular marker technology has been widely applied, utilizing markers closely linked to resistance genes for assisted breeding is a simple, fast, and time-saving breeding method. In this study, a total of nine molecular markers related to potato PVY resistance were collected and used to screen potato germplasm. Markers RYSC3 and YES3-3B were able to amplify the target band of the expected size, with clear and good repeatability. The amplification results of the other seven marker primers had poor repeatability or could not amplify the target band. RYSC3 and YES3-3B were widely used for the detection of *Ry_adg_* and *Ry_sto_* (Fulladolsa et al., 2015; Jiang et al., 2022). In this study, RYSC3 was detected in the genomic DNA of 125 potato accessions, with a detection rate of 90.579%; YES3-3B was detected in the genomic DNA of 121 potato accessions with a detection rate of 87.681%, indicating that most of the tested germplasm contains PVY resistance markers, RYSC3 and YES3-3B.

Our results also indicate that most of the tested germplasm accessions containing markers, RYSC3 and YES3-3B, were identified as resistant (HR, R or MR) using artificial inoculation, indicating that molecular markers are accurate in identifying PVY-resistant materials. Among the germplasm identified as susceptible (S or HS) by artificial inoculation, only 10.390% of the them did not contain marker RYSC3, 12.987% of them did not contain marker YES3-3B, but the remaining germplasm contained these markers. This may be because the accessions with these markers still exhibit severe symptoms after inoculation, thus being judged as PVY-susceptible germplasm. Some accessions have been identified as PVY-resistant materials based on artificial inoculation (Huang, 2009), while markers RYSC3 and YES3-3B have not been detected in their genomic DNA. This might be due some accessions being able to tolerate virus infection and proliferation and exhibiting little impact on plant growth and yield, or their genomic DNA containing other resistance markers apart from RYSC3 and YES3-3B.

Based on genetic distance and cluster analysis results, 16 accessions with relatively long genetic distance from other accessions which had a wide range of sources were screened. Meanwhile, based on the PVY resistance evaluation results of potato germplasm, 14 accessions with better PVY resistance were screened. Based on the comprehensive analysis of genetic distance and PVY resistance, six accessions, No. 122, 79, 38, 61, 108, and 132, were identified to have both PVY-resistance and distant genetic distance with other accessions. They were valuable resources for PVY resistance breeding of potato. Using these accessions in developing new varieties or improving existing varieties can not only improve the PVY resistance of potato varieties, but also contribute to enhancing the genetic diversity of potato varieties.

In addition, the distribution of high-resistant materials (HR) covers the Netherlands, China and the United States, the high-resistant materials of disease resistance (R) are distributed in China, Canada, the Netherlands and the United Kingdom, and the medium-resistant materials (MR) are widely distributed, coming from 10 countries such as the United Kingdom, the Netherlands and China. This distribution shows the existence of highly-resistant, disease-resistant and medium-resistant materials that can be cultivated or screened in multiple regions, and is not limited to a specific region. This means that disease resistance is an important breeding goal in these areas. It is worth noting that China has more highly resistant, resistant and medium resistant materials, with 2, 5 and 19 numbers respectively from this region. This may imply that China has made remarkable achievements in breeding for PVY resistance, or that China faces more severe disease pressure, so the need for disease resistance is more urgent. The Netherlands is also a significant source of highly resistant, disease resistant and medium resistant materials, with 2, 3 and 9 accessions, respectively from this region. In addition, it is important to validate whether the highly resistant genotypes can retain the resistance in the PVY hot spots under different agro-climatic conditions across the globe.

## 5 Conclusion

A detailed genetic cluster analysis of 138 potato germplasm accessions from 16 countries using highly polymorphic SSR markers, showed wide genetic diversity among the germplasm. This discovery not only reflects the extensive genetic background of potato germplasm, but also provides a solid material basis for subsequent genetic improvement and breeding work. Comparison between genetic cluster analysis and PVY resistance revealed the accessions with specific PVY resistance types relatively distributed in the same genetic cluster, which provides important clues, further revealing the genetic mechanism of PVY resistance genes. Through comprehensive evaluation of the genetic distance and PVY resistance, we have successfully screened six genotypes that have a distant genetic relationship with other germplasm accessions and exhibit stable and reliable PVY resistance. These materials can be used as valuable sources for PVY-resistance breeding, and have profound strategic significance for cultivating new disease resistant potato varieties, improving existing varieties, and enriching the genetic variability of breeding populations. By utilizing these resources, we can more effectively address the threat of PVY virus to the potato industry, increase potato production and quality, and ensure global food security.

In summary, this study not only enriches the genetic diversity database of potato germplasm, but also provides new germplasm and strategies for potato resistance breeding. We believe that these achievements will provide important references for the research and utilization of potato germplasm, injecting new vitality into the sustainable development of the potato industry.

## Data Availability

The original contributions presented in the study are included in the article/**Supplementary Material**, further inquiries can be directed to the corresponding authors.

## References

[B1] Asama K. Ito H. Murakami N. Itoh T. (1982). New potato variety “Konafubuki. Bull. Hokkaido Prefect. Agric. Exp. Stn. 6, 75–84.

[B2] Bai L. Guo H.-C. (2017). Molecular marker-assisted screening of potato polyclonal antibody parents. Mol. Plant Breed. 15, 200–212.

[B3] Caruana B. M. Rodoni B. C. Constable F. Slater A. T. Cogan N. O. I. (2021). Genome enhanced marker improvement for potato virus Y disease resistance in potato. Agronomy 11, 832. doi: 10.3390/agronomy11050832

[B4] Doyle J. J. Doyle J. L. (1990). Isolation of plant DNA from fresh tissue. Focus 12, 13–15.

[B5] Duan S. G. (2017). Evaluation of genetic diversity of potato germplasm resources and genetic analysis of important traits. Chin. Acad. Agric. Sci. 01, 109.

[B6] Elison L. G. Hall G. D. Novy G. R. Whitworth J. (2020). Development and application of a multiplex marker assay to detect PVY resistance genes in *Solanum tuberosum* . Am. J. Potato Res. 97, 289–296. doi: 10.1007/s12230-020-09777-1

[B7] Flis B. Hennig J. Strzelczyk D. Gebhardt G. Marczewski W. (2005). The *Ry-f_sto_ * gene from *Solanum stloniferum* for extreme resistant to Potato virus Y maps to potato chromosome XII and is diagnosed by PCR marker GP122_718_ in PVY resistant potato cultivars. Mol. Breed. 15, 95–101. doi: 10.1007/s11032-004-2736-3

[B8] Fulladolsa A. C. Navarro F. M. Kota R. Severson K. Palta J. P. Charkowski A. (2015). Application of marker assisted selection for potato virus Y resistance in the University of Wisconsin potato breeding program. Am. J. Potato Res. 92, 444–450. doi: 10.1007/s12230-015-9431-2

[B9] Grech-Baran M. Witek K. Szajko K. Witek A. I. Morgiewicz K. Wasilewicz-Flis I.. (2020). Extreme resistance to *Potato virus Y* in potato carrying the *Ry_sto_ * gene is mediated by a TIR-NLR immune receptor. Plant Biotech. J. 18, 655–667. doi: 10.1111/pbi.13230 PMC7004898 31397954

[B10] Hämäläinen J. H. Watanabe K. N. Valkonen J. P. T. Arihara A. Plaisted R. L. Pehu E.. (1997). Mapping and marker-assisted selection for a gene for extreme resistance to potato virus Y. Theor. Appl. Genet. 94, 192–197. doi: 10.1007/s001220050399

[B11] Han Q. Sun X. L. Lu Y. Tian D. Shi B. Shen X. F. (2020). Construction of DNA fingerprinting database of 50 waxy maize inbred lines using SNP markers. J. Shanghai Agric. Sci. 36, 15–19. doi: 10.15955/j.issn1000-3924.2020.03.03

[B12] Hosseini A. Massumi H. Heydarnejad J. Pour A. H. Varsani A. (2011). Characterisation of potato virus Y isolates from Iran. Virus Genes 42, 128–140. doi: 10.1007/s11262-010-0546-8 21082231

[B13] Huang M. J. (2009). Screening and evaluation of PVY resistant materials in wild potato (Northeast Agricultural University) 02, 67.

[B14] Jiang W. Lu L. L. Bao L. X. Li X. F. Yang S. Y. Yang R. K.. (2022). Research on molecular marker screening of germplasm resources resistant to potato Y virus. Chin. Veg. 3, 16–22. doi: 10.19928/j.cnki.1000-6346.2022.1013

[B15] Kasai K. Morikawa Y. Sorri V. A. Valkonen J. P. Gebhardt C. Watanabe K. N. (2000). Development of SCAR markers to the PVY resistance gene Ryadg based on a common feature of plant disease resistance genes. Genome 43, 1–8. doi: 10.1139/g99-092 10701106

[B16] Kehoe M. A. Jones R. A. C. (2016). Improving potato virus Y strain nomenclature: Lessons from comparing isolates obtained over a 73-year period. Plant Pathol. 65, 322–333. doi: 10.1111/ppa.12404

[B17] Kleinwechter U. Gastelo M. Ritchie J. Nelson G. Asseng S. (2016). Simulating cultivar variations in potato yields for contrasting environments. Plant Pathol. 145, 51–63. doi: 10.1016/j.agsy.2016.02.011

[B18] Kolychikhina M. S. Beloshapkina O. O. Phiri C. K. (2021). Change in potato productivity under the impact of viral diseases. IOP Conf. Ser.: Earth Environ. Sci. 663, 12035. doi: 10.1088/1755-1315/663/1/012035

[B19] Kreuze J. Souza-Dias J. Jeevalatha A. Figueira A. Valkonen J. Jones R. (2020). “Viral diseases in potato,” in The potato crop. Eds. Campos H. Ortiz O. (Springer, Cham), 389–430. doi: 10.1007/978-3-030-28683-5_11

[B20] Li T. Deng G. B. Tang Y. Y. Su Y. Wang J. H. Liang J. J.. (2019). Mapping the dominant QTL of wheat spikelet number based on high-density genetic linkage mapping. Abstract 10th Natl. Congress Wheat Genomics Mol. Breed. 1, 157–158. doi: 10.26914/c.cnkihy.2019.021041

[B21] Li J. W. Li H. Liu Z. W. Wang Y. X. Chen Y. Yang N.. (2023). Molecular markers in tea plant (Camellia sinensis): Applications to evolution, genetic identification, and molecular breeding. Plant Physiol. Biochem. 198, 107704. doi: 10.1016/j.plaphy.2023.107704 37086694

[B22] Li J. Li M. Wang F. Ye G. J. Zhou Y. Wang J. (2017). Application of RYSC3 and Rxsp molecular markers in potato breeding for disease resistance. Mol. Plant Breed. 15, 4040–4046. doi: 10.13271/j.mpb.015.004040

[B23] Lin S. Dewey R. E. Wang R. Yu J. Long M. Zhang J.. (2021). Discovery of a naturally-occurring allele of eIF4E1.S in Nicotiana tabacum and development of a co-dominant marker. Euphytica 217, 122. doi: 10.1007/s10681-021-02876-1

[B24] Liu Y. Q. Tian Z. M. Qi L. P. Gong X. C. Feng Y. Zhang Y. S.. (2022). Construction of fingerprint and genetic diversity analysis of 20 potato varieties (lines). Jiangsu Agric. Sci. 50, 41–46. doi: 10.15889/j.issn.1002-1302.2022.21.006

[B25] Lou S. Yang M. Xing J. Zhai L. X. Wang H. Liu C. S.. (2023). Potato germplasm resources antiviral marker-assisted selection. Crops 4, 65–70. doi: 10.16035/j.iSSN.1001-7283.2023.04.010

[B26] Mao Y. Z. (2009). A study on the identification of potato plant resistance by artificial inoculation with PVY virus. Agric. Sci. Technol. Commun. 5, 59–60.

[B27] Mori K. Sakamoto Y. Mukojima N. Tamiya S. Nakao T. Ishii T.. (2011). Development of a multiplex PCR method for simultaneous detection of diagnostic DNA markers of five disease and pest resistance genes in potato. Euphytica 65, 347–355. doi: 10.1007/s10681-011-0381-6

[B28] Munoz F. J. Plaisted R. L. Thurston H. D. (1975). Resistance to potato virus Y in *Solanum tuberosum* spp. andigena. Am. Potato J. 52, 107–115. doi: 10.1007/BF02852043

[B29] Saidi A. Hajibarat Z. (2021). Approaches for developing molecular markers associated with virus resistances in potato (Solanum tuberosum). J. Plant Dis. Prot. 128, 649–662. doi: 10.1007/s41348-021-00440-3

[B30] Sato M. Nishikawa K. Komura K. Hosaka K. (2006). Potato virus Y resistance gene, Rychc, mapped to the distal end of potato chromosome 9. Euphytica 149, 367–372. doi: 10.1007/s10681-006-9090-y

[B31] Shah R. A. Bakshi P. Jasrotia A. Itoo H. Padder B. A. Gupta R.. (2023). Morphological to molecular markers: Plant genetic diversity studies in walnut (Juglans regia L.) - A review. Res. Sq. 65, 1499–1511. doi: 10.21203/rs.3.rs-1764160/v1

[B32] Slater A. T. Schultz L. Lombardi M. Rodoni B. C. Bottcher C. Cogan N. O. I.. (2020). Screening for resistance to PVY in Australian potato germplasm. Genes 11, 429. doi: 10.3390/genes11040429 32316258 PMC7230960

[B33] Song G. Guo Y. S. Rao L. X. Zhou X. P. Wu J. X. (2016). Preparation and detection application of monoclonal antibodies against potato Y virus. J. Zhejiang Univ. Agric. Life Sci. 42, 517–526.

[B34] Song Y. S. Leonard H. Günther S. Hartl L. Wenzel G. Schwarzfischer A. (2005). Mapping of extreme resistance to PVY (*Ry_sto_ *) on chromosome XII using anther-culture-derived primary dihaploid potato lines. Theor. Appl. Genet. 111, 879–887. doi: 10.1007/s00122-005-0010-7 16044270

[B35] Song Y. S. Schwarzfischer A. (2008). Development of STS markers for selection of extreme resistance (Rysto) to PVY and maternal pedigree analysis of extremely resistant cultivars. Am. J. Potato Res. 85, 392–393. doi: 10.1007/s12230-008-9044-0

[B36] Srivastava R. K. Talla A. Swain D. K. Panda R. K. (2019). Quantitative approaches in adaptation strategies to cope with increased temperatures following climate change in potato crop. Potato Res. 62, 175–191. doi: 10.1007/s11540-018-9406-z

[B37] Tang D. Zhang K. Lv C. Xie D. Fu T. Wang J. (2016). Construction of linkage map and QTL mapping of starch traits of sweet potato based on EST-SSR markers. Chin. J. Agric. Sci. 23, 4488–4506.

[B38] Tian J. Chen J. Ye X. Chen S. (2016). Health benefits of the potato affected by domestic cooking: A review. Food Chem. 202, 165–175. doi: 10.1016/j.foodchem.2016.01.120 26920281

[B39] Valkonen J. P. T. Wiegmann K. Hämäläinen J. H. Marczewski W. Watanabe K. N. (2007). Evidence for utility of the same PCR-based markers for selection of extreme resistance to potato virus Y controlled by Ry_sto_ of Solanum stoloniferum derived from different sources. Ann. Appl. Biol. 152, 121–130.

[B40] Wang P. Li F. D. Guo T. S. Dou J. H. Jie W. Q. Luo Z. X.. (2019). Genetic diversity analysis of SSR marked potato cultivars. Chin. Potato 33, 257–266.

[B41] Wang Y. Rashid M. A. R. Li X. Yao C. Lu L. Bai J.. (2019). Collection and evaluation of genetic diversity and population structure of potato landraces and varieties in China. Front. Plant Sci. 10. doi: 10.3389/fpls.2019.00139 PMC6393402 30846993

[B42] Yang Z. P. (2006). *Research on genetic diversity of germplasm resources of potato (*Solanum tuberosum *L.)* . Southwest Univ. 12, 60.

[B43] Zhang Z. Wang X. Guan J. Zhang D. Li Z. Zhang M.. (2024). Molecular markers and candidate genes of plant height traits in upland cotton identified by single-locus and multi-locus genome-wide association study. Crop Sci. 1, 1743–1755. doi: 10.1002/csc2.21248

[B44] Zhao X. T. (2012). Establishment and application of marker-assisted selection for PVY and PVX resistance in potato (*Solanum tuberosum* L.). Chin. Acad. Agric. Sci. 10, 79.

[B45] Zhao L. K. (2020). Genetic diversity analysis and fingerprint construction of 115 registered sweet potato varieties in China. Chin. Acad. Agric. Sci 01, 59.

[B46] Zhu Y. F. (2013). Research on molecular marker identification and fingerprint construction of crop varieties. Zhejiang Univ. 12, 124.

