# Supplementary material for: Genetic profiling and PVY resistance identification of potato germplasm resources
Source: Front Plant Sci. 2024 Sep 20;15:1444281. doi: 10.3389/fpls.2024.1444281 (PMC11450225; doi:10.3389/fpls.2024.1444281)
Supplement: Supplementary file 1 [file DataSheet1.docx]

**Supplementary Table 1.** Information of potato germplasm resources used in this study.

| Resource Number | Resource Name | Source | Type |
| --- | --- | --- | --- |
| 1 | XN723 | China | - |
| 2 | XN726 | China | - |
| 3 | XN727 | China | - |
| 4 | XN746 | China | - |
| 5 | XN749 | China | - |
| 6 | XN750 | China | - |
| 7 | XN751 | China | - |
| 8 | Minshu | China | Fresh food varieties |
| 9 | Youjin | China | Combination of fresh and fried food |
| 10 | Atlantic | USA | Variety of fried chips |
| 11 | Xinjia No.2 | China | Fresh food and starch processing |
| 12 | XN806 | China | - |
| 13 | XN841 | China | - |
| 14 | Jia 1219 | Canada | Processing varieties of French fries |
| 15 | XN845 | China | - |
| 16 | Jia 231 | Canada | Processing varieties of French fries |
| 17 | Jia 1890 | Canada | Processing varieties of French fries |
| 18 | Jia 2117 | Canada | Processing varieties of French fries |
| 19 | 2223 | Canada | Processing varieties of French fries |
| 20 | 2228 | Canada | Processing varieties of French fries |
| 21 | XN854 | China | - |
| 22 | 2148 | Canada | Processing varieties of French fries |
| 23 | mantana Ranger | Canada | Processing varieties of French fries |
| 24 | Shepody-1 | Canada | Processing varieties of French fries |
| 25 | zwnova70r | Canada | Processing varieties of French fries |
| 26 | Ranger Russet | USA | Processing varieties of French fries |
| 27 | KENNEBEC | USA | Processing varieties of French fries |
| 28 | XN879 | China | - |
| 29 | XN907 | China | - |
| 30 | Biyin No.4 | Belgium | - |
| 31 | Bibiao104 | Belgium | - |
| 32 | Bibiao105 | Belgium | - |
| 33 | Bikang Bionica | Belgium | - |
| 34 | XN923 | China | - |
| 35 | fianna | Holland | - |
| 36 | fontane | Holland | - |
| 37 | Lady blanca | Holland | - |
| 38 | Sarpo mira | Denmark | - |
| 39 | XN931 | China | - |
| 40 | Ri6-1 | Japan | - |
| 41 | Ri6-2 | Japan | - |
| 42 | Ri6-3 | Japan | - |
| 43 | Ying 1 | UK | - |
| 44 | Nuowei 1 | Norway | - |
| 45 | XN940 | China | - |
| 46 | lionheart | UK | - |
| 47 | XN942 | China | - |
| 48 | M2 | USA | - |
| 49 | M3 | USA | - |
| 50 | XN948 | China | - |
| 51 | XN950 | China | - |
| 52 | KE | Belgium | - |
| 53 | Shepody-2 | Belgium | - |
| 54 | France line | Belgium | - |
| 55 | XN957 | China | - |
| 56 | Chaoyin No.4 | NK | - |
| 57 | Heyin No.1 | Holland | - |
| 58 | Heyin No.2 | Holland | - |
| 59 | innorator | Holland | - |
| 60 | Actrice | Holland | - |
| 61 | Desiree | Holland | Fresh food varieties |
| 62 | XN970 | China | - |
| 63 | Jeanntte | Holland | - |
| 64 | D05-47-03 | Holland | - |
| 65 | Agria | Holland | - |
| 66 | XN975 | China | - |
| 67 | ACM | Holland | - |
| 68 | Fenlan-1 | Norway | - |
| 69 | XN979 | China | - |
| 70 | XN980 | China | - |
| 71 | Ranger | USA | - |
| 72 | Organic Dabai | Japan | - |
| 73 | XN987 | China | - |
| 74 | Organic Red Andes | Japan | - |
| 75 | XN989 | China | - |
| 76 | Organic Nanjue | Japan | - |
| 77 | Organic Beiming | Japan | - |
| 78 | Burbank No.2 | USA | Genetically modified varieties |
| 79 | XN995 | Russia | - |
| 80 | XN996 | China | - |
| 81 | XN997 | China | - |
| 82 | Daihao 2 | USA | - |
| 83 | XN999 | China | - |
| 84 | HZ | Holland | - |
| 85 | HY | Holland | - |
| 86 | HL | Holland | - |
| 87 | RUNSHI | Switzerland | - |
| 88 | Minyin | China | - |
| 89 | XN1005 | China | - |
| 90 | XN1007 | China | - |
| 91 | Holland No.3 | Holland | - |
| 92 | Holland No.4 | Holland | - |
| 93 | Holland No.5 | Holland | - |
| 94 | XN1011 | China | - |
| 95 | Holland No.7 | Holland | - |
| 96 | Holland No.8 | Holland | - |
| 97 | Holland No.9 | Holland | - |
| 98 | Heyin No.10 | Holland | - |
| 99 | XN1017 | China | - |
| 100 | Heyin No.12 | Holland | - |
| 101 | XN1019 | China | - |
| 102 | XN1021 | China | - |
| 103 | Heyin No.17 | Holland | - |
| 104 | Heyin No.18 | Holland | - |
| 105 | XN1025 | China | - |
| 106 | Heyin No.20 | Holland | - |
| 107 | Heyin No.21 | Holland | - |
| 108 | Biyin No.22 | Belgium | - |
| 109 | XN1029 | China | - |
| 110 | XN1031 | China | - |
| 111 | XN1032 | China | - |
| 112 | Suyin No.1 | UK | - |
| 113 | Suyin No.2 | UK | - |
| 114 | Suyin No.6 | UK | - |
| 115 | Suyin No.10 | UK | - |
| 116 | XN1043 | China | - |
| 117 | Suyin No.12 | UK | - |
| 118 | XN1046 | China | - |
| 119 | Riyin No.1 | Japan | - |
| 120 | Wuyin No.1 | Ukraine | - |
| 121 | Aiyin No.1 | Ireland | - |
| 122 | Moyin No.1 | Morocco | - |
| 123 | XN1051 | China | - |
| 124 | Lv No.1 | Holland | - |
| 125 | Suyin | UK | - |
| 126 | Hanguo No.1 | SK | - |
| 127 | XN1055 | China | - |
| 128 | Z1047 | China | - |
| 129 | Z1048 | China | - |
| 130 | Z1049 | China | - |
| 131 | Z1050 | China | - |
| 132 | Z173 | China | - |
| 133 | Z456 | China | - |
| 134 | Z466 | China | - |
| 135 | Z470 | China | - |
| 136 | Yu potato No.5 | China | - |
| 137 | brl-Z | China | - |
| 138 | Z937 | China | - |

**Supplementary Table 2.** Primer pairs of 41 SSR markers

| SSR marker | Primer sequences 5’~3’ | Primer sequences 5’~3’ |
| --- | --- | --- |
| St-1 | GGAGTCAAGTTTGCTCACAATC | CACCCTCCAACCCCATATC |
| St-2 | TTCGTTGCTTACCTACTA | CCCAAGATTACACATTC |
| St-16 | GTTCATGATTGTGAATGCTC | ATGACTCAACCCAAATG |
| St-24 | AATCGGTGGATAATGTGAATGC | ATGCTTGCCATGTGATGTGT |
| St-26 | GACACGTTCACATAAA | AGAAGAATAAGCAAAGCAA |
| St-28 | CATTACCTCCATTGCTA | GTCTCGTACTTTTCAT |
| St-30 | GAAAACCTTGAGAGGGCAAAGAGT | ATTCACGAGCATTGTCAAGCGTTC |
| St-42 | AATTTAACTTAGAAGATTAGTCTC | ATTTGTGGGTATGATA |
| St-49 | GTACACAGCAAAATAGCAAG | TAGACACTCTCACATCCACT |
| St-50 | ACCATCCTAAACCAGATAT | GGGGAGAGGAGTAAAACAT |
| St-52 | TCAACCTATTATTTTGAGTCG | CATACGCACGCACGTACAC |
| St-68 | TTCAGAGACATCATGGCAACTT | ATCCTTCATCAGAGGAGAAATCC |
| St-71 | GGCTGCCAGGATTATTGTGTTC | GATGTAAACACGTGTGTGTG |
| St-73 | AATGTCATTCGTTACTTCCC | AGTCAAGGGAGTAAACAAAG |
| St-74 | GGACAATCCATAAATCTCAG | AGCAGCAGCAGCCTCTCCTA |
| St-76 | ATTTCGTTGCTTACCTACTA | AACCCAAGATTACCACATTC |
| St-78 | GGAAGATTTGTAGGTTCAA | AAAGTGAAACTTTCCTAGCATG |
| St-80 | ATTTGATTGAAGAACTTATATAGAA | CACAAACAAAATACTGTTAACTCA |
| St-83 | TCCCCTTGGCATTTTTTCTCC | TTTAGGGGTGGGGGGGGGGGGGAGGTTGG |
| St-104 | TTCGGAATTACCTCTGCC | AAAAAAAGAACGACGCACG |
| St-107 | TCTCACCCAGCGGAACAT | AAGCTGCGGAAGTGATTTTG |
| St-109 | ACAGCTTACTCTGCTCCAACAA | TGACGGCGTTTTACAAC |
| St-138 | GGCAAAATTCATTCAACTC | GTCTGCTTCCCGGAAATTTT |
| St-140 | GCTATTGTCAGATATATACG | GCCATGCACTAATTTTGA |
| St-142 | CTATTCCGATCCAGCAACAA | CTGACCACCCTTCGCTACAT |
| St-144 | TGAGAATTGTGGAGGCAAGA | GCATATAAATTCAAAGATCACGAC |
| St-146 | AGACCTTACGAACATATCCC | TACAACCATAGAGCACAGCA |
| St-148 | TCTTCTTGATGACAGCTTCG | ACCTCAGATAGTTGCCATGTCA |
| St-150 | TCAAAACCCCAAATCAC | AAATGATTAGCTTCCCCGCA |
| St-151 | AAGCGTGAACTATCCGGTACT | ACCCATATTGTGATGTACCA |
| St-153 | TCGCTTCTTACTTGTGTGGC | GATACAGCTACTGCAACTCCAC |
| St-156 | CATTTTCTCCAGTAACTAACCA | TTAGCTCCTATTTGAATCCC |
| St-176 | CAACCAATTTCGAGGAC | TGTCCCAAATCGTCTGGC |
| St-178 | TTTTACGGTGAAGCCACTCC | CTTGGGTTGCCAGTTTG |
| St-180 | ACCATAACCATCATCGTCTAC | GATAGTGTTGGTGTGTTC |
| St-182 | ACAGGAATCACACCTGCACA | TTCAACATCCGCCTGTCATA |
| St-192 | CCACTTCCTCCACTTCCAAA | CCATGTTCACAACTAGA |
| St-213 | CAAGAACACAAGAGATTTTCA | TGGCGAATGTGAGAAACAAA |
| St-215 | GGACAACCAAGTGAGCAACA | TGAGGAGAAAGGCACACAAA |
| St-218 | CTCTGTTTCTAATCGGCCGTA | AAGCGTTGGCCACCGCCA |
| St-255 | CCCATAATACTGTGATGAGCA | GAATGTAGGGAACATGCATGA |

**Supplementary Table 3.** Standard for Grading Evaluation of Resistance to Potato Virus

| Resistance level | Disease index | Disease resistant types |
| --- | --- | --- |
| 0 | DI=0 | Immunology (I) |
| 1 | 0<DI ≤ 5 | High resistance (HR) |
| 2 | 5<DI ≤ 20 | Disease resistance (R) |
| 3 | 20<DI ≤ 35 | Medium resistance (MR) |
| 4 | 35<DI ≤ 60 | Infected (S) |
| 5 | 60<DI ≤ 100 | High sensitivity (HS) |

**Supplementary Table 4.** Molecular markers linked to PVY resistance genes in potato

| Marker | Source species | Gene | Chromosome | Primer Sequence | Annealing temperature (℃) | Amplification product length (bp) | Type of marker (restriction endonuclease) |
| --- | --- | --- | --- | --- | --- | --- | --- |
| RYSC3 | *S. Andigena* | Ry_adg_ | XI | ATACACTCATCTAATTGATGG | 60 | 321 | SCAR |
|  |  |  |  | AGGATATACGGCATTTCCGA |  |  |  |
| M45 | *S. Andigena* | Ry_adg_ | XI | GACTGCGTACATGCAGCT | 60 | 493 | AFLP |
|  |  |  |  | GATGAGTTCTGAGTAGGA |  |  |  |
| YES3-3B | *S. Stoloniferum* | Ry_sto_ | XI | TAACTCAAGCGGAATAACCC | 55 | 284 | STS |
|  |  |  |  | CATGAGATTGCCTTTGGTTA |  |  |  |
| YES3-3A | *S. Stoloniferum* | Ry_sto_ | XII | TAACTCAAGCGGAATAACCC | 53 | 341 | STS |
|  |  |  |  | AATTCACCTGTTTTACATGCTTCTTGTG |  |  |  |
| STM0003 | *S. Stoloniferum* | Ry_sto_ | XII | GGAGAATCATAACAACCAG | 58 | 111 | SSR |
|  |  |  |  | AATTGTAACTCTGTGTGTGTGTGTG3 |  |  |  |
| SCARysto4 | *S. Stoloniferum* | Ry_sto_ | XII | ATTTCGTTCGCCTCTCTCCT | 54 | 110 | SCAR |
|  |  |  |  | TCATCACCCTAACAATACAA |  |  |  |
| GP122-718 | *S. Stoloniferum* | Ry_sto_ | XII | TATTTTAGGTACTTTTTTTTTCTTA | 53 | 751 | CAPS (EcoRV) |
|  |  |  |  | GCACTCAATAGCCCTTCTT |  |  |  |
| GP122-546 | *S. Stoloniferum* | Ry_sto_ | XII | TATTTTAGGGGTACTTCTTTCTTATGTT | 56 | 614 | CAPS (EcoRV and Mboi.) |
|  |  |  |  | CTGTCAAAAAATTCACTTGCTTCATAACTAC |  |  |  |
| Ry186 | *S. Konafubuki* | Ry_chc_ | IX | TGGTAGGGATTTTCCTTAGA | 55 | 587 | STS |
|  |  |  |  | GCAAATCCTAGGTTATCAACTCA |  |  |  |

**Supplementary Table 5.** Genetic distances between some germplasms

| Resource No. | 1 | 2 | 3 |  | 61 | 62 | 63 | 64 |  | 101 | 102 |  | 116 |  | 137 | 138 |
| --- | --- | --- | --- | --- | --- | --- | --- | --- | --- | --- | --- | --- | --- | --- | --- | --- |
| 1 | 0 |  |  |  |  |  |  |  |  |  |  |  |  |  |  |  |
| 2 | 0.129 | 0 |  |  |  |  |  |  |  |  |  |  |  |  |  |  |
| 3 | 0.176 | 0.155 | 0 |  |  |  |  |  |  |  |  |  |  |  |  |  |
|  |  |  |  |  |  |  |  |  |  |  |  |  |  |  |  |  |
| 61 | 0.221 | 0.238 | 0.303 |  | 0 |  |  |  |  |  |  |  |  |  |  |  |
| 62 | 0.175 | 0.15 | 0.19 |  | 0.3 | 0 |  |  |  |  |  |  |  |  |  |  |
| 63 | 0.197 | 0.177 | 0.258 |  | 0.248 | 0.25 | 0 |  |  |  |  |  |  |  |  |  |
| 64 | 0.183 | 0.157 | 0.272 |  | 0.254 | 0.211 | 0.209 | 0 |  |  |  |  |  |  |  |  |
|  |  |  |  |  |  |  |  |  |  |  |  |  |  |  |  |  |
| 101 | 0.153 | 0.145 | 0.136 |  | 0.215 | 0.202 | 0.154 | 0.258 |  | 0 |  |  |  |  |  |  |
| 102 | 0.166 | 0.109 | 0.191 |  | 0.273 | 0.198 | 0.199 | 0.182 |  | 0.16 | 0 |  |  |  |  |  |
|  |  |  |  |  |  |  |  |  |  |  |  |  |  |  |  |  |
| 116 | 0.264 | 0.244 | 0.248 |  | 0.23 | 0.236 | 0.252 | 0.275 |  | 0.261 | 0.247 |  | 0 |  |  |  |
|  |  |  |  |  |  |  |  |  |  |  |  |  |  |  |  |  |
| 137 | 0.191 | 0.15 | 0.259 |  | 0.246 | 0.207 | 0.132 | 0.179 |  | 0.191 | 0.194 |  | 0.196 |  | 0 |  |
| 138 | 0.233 | 0.198 | 0.289 |  | 0.292 | 0.255 | 0.199 | 0.19 |  | 0.197 | 0.184 |  | 0.269 |  | 0.15 | 0 |

**Supplementary Table 6.** Disease indexes of different potato germplasm resources.

| Resource Number | Disease Index | Multiple Comparison | Resistance |  | Resource Number | Disease Index | Multiple Comparison | Resistance |
| --- | --- | --- | --- | --- | --- | --- | --- | --- |
| 44 | 92.76 | a | HS |  | 1 | 40.08 | ghi | S |
| 55 | 87.27 | ab | HS |  | 65 | 40.08 | ghi | S |
| 56 | 86.86 | ab | HS |  | 81 | 40.06 | ghi | S |
| 86 | 86.78 | ab | HS |  | 18 | 40.04 | ghi | S |
| 30 | 86.78 | ab | HS |  | 25 | 39.95 | ghi | S |
| 14 | 86.75 | ab | HS |  | 80 | 39.75 | ghi | S |
| 85 | 86.75 | ab | HS |  | 66 | 39.75 | ghi | S |
| 6 | 86.67 | ab | HS |  | 51 | 39.61 | ghij | S |
| 98 | 86.64 | ab | HS |  | 117 | 33.74 | hijk | MR |
| 21 | 86.64 | ab | HS |  | 35 | 33.47 | hijk | MR |
| 74 | 80.29 | abc | HS |  | 71 | 33.4 | hijk | MR |
| 138 | 80.19 | abc | HS |  | 101 | 33.4 | hijk | MR |
| 137 | 80.14 | abc | HS |  | 97 | 33.38 | hijk | MR |
| 22 | 80.11 | abc | HS |  | 2 | 33.34 | hijk | MR |
| 77 | 80.08 | abc | HS |  | 62 | 33.34 | hijk | MR |
| 50 | 80.04 | abc | HS |  | 38 | 33.27 | hijk | MR |
| 29 | 80.01 | abc | HS |  | 124 | 33.11 | hijk | MR |
| 7 | 79.98 | abc | HS |  | 64 | 32.74 | hijkl | MR |
| 41 | 79.68 | abc | HS |  | 116 | 32.61 | hijkl | MR |
| 109 | 73.41 | bcd | HS |  | 15 | 27.07 | ijklm | MR |
| 48 | 73.37 | bcd | HS |  | 42 | 27.01 | ijklm | MR |
| 131 | 73.31 | bcd | HS |  | 95 | 27 | ijklm | MR |
| 57 | 66.84 | cde | HS |  | 78 | 26.88 | ijklm | MR |
| 76 | 66.75 | cde | HS |  | 122 | 26.86 | ijklm | MR |
| 75 | 66.75 | cde | HS |  | 73 | 26.81 | ijklm | MR |
| 102 | 66.71 | cde | HS |  | 5 | 26.81 | ijklm | MR |
| 16 | 66.6 | cde | HS |  | 33 | 26.79 | ijklm | MR |
| 52 | 66.55 | cde | HS |  | 108 | 26.77 | ijklm | MR |
| 94 | 66.55 | cde | HS |  | 67 | 26.77 | ijklm | MR |
| 11 | 66.55 | cde | HS |  | 3 | 26.75 | ijklm | MR |
| 32 | 66.51 | cde | HS |  | 61 | 26.75 | ijklm | MR |
| 54 | 66.48 | cde | HS |  | 10 | 26.71 | ijklm | MR |
| 106 | 66.45 | cde | HS |  | 40 | 26.71 | ijklm | MR |
| 93 | 66.42 | cde | HS |  | 128 | 26.71 | ijklm | MR |
| 134 | 66.29 | cde | HS |  | 92 | 26.65 | ijklm | MR |
| 87 | 66.28 | cde | HS |  | 43 | 26.64 | ijklm | MR |
| 58 | 66.27 | cde | HS |  | 8 | 26.62 | ijklm | MR |
| 68 | 60.48 | def | HS |  | 119 | 26.61 | ijklm | MR |
| 113 | 60.15 | def | HS |  | 82 | 26.6 | ijklm | MR |
| 23 | 60.12 | def | HS |  | 45 | 26.55 | ijklm | MR |
| 107 | 60.11 | def | HS |  | 120 | 26.52 | ijklm | MR |
| 26 | 60.04 | def | HS |  | 12 | 26.49 | ijklm | MR |
| 99 | 59.95 | def | S |  | 79 | 26.46 | ijklm | MR |
| 90 | 59.94 | def | S |  | 28 | 26.42 | ijklm | MR |
| 83 | 59.91 | def | S |  | 69 | 26.27 | ijklm | MR |
| 63 | 59.75 | def | S |  | 133 | 20.99 | jklmn | MR |
| 125 | 53.48 | efg | S |  | 115 | 20.43 | klmn | MR |
| 72 | 53.47 | efg | S |  | 84 | 20.41 | klmn | MR |
| 121 | 53.3 | efg | S |  | 19 | 20.41 | klmn | MR |
| 53 | 46.95 | fgh | S |  | 88 | 20.21 | klmn | MR |
| 110 | 46.82 | fgh | S |  | 135 | 20.14 | klmn | MR |
| 47 | 46.82 | fgh | S |  | 129 | 20.09 | klmn | MR |
| 17 | 46.79 | fgh | S |  | 13 | 20.07 | klmn | MR |
| 60 | 46.75 | fgh | S |  | 9 | 19.98 | klmn | R |
| 36 | 46.75 | fgh | S |  | 130 | 19.95 | klmn | R |
| 112 | 46.75 | fgh | S |  | 118 | 19.95 | klmn | R |
| 46 | 46.74 | fgh | S |  | 24 | 19.94 | klmn | R |
| 49 | 46.74 | fgh | S |  | 132 | 14.24 | lmn | R |
| 127 | 46.7 | fgh | S |  | 59 | 13.41 | mn | R |
| 123 | 46.69 | fgh | S |  | 114 | 13.38 | mn | R |
| 126 | 46.63 | fgh | S |  | 104 | 7.18 | n | R |
| 37 | 46.51 | fgh | S |  | 103 | 6.92 | n | R |
| 70 | 40.3 | ghi | S |  | 136 | 6.82 | n | R |
| 100 | 40.21 | ghi | S |  | 20 | 6.56 | n | R |
| 89 | 40.18 | ghi | S |  | 91 | 4.96 | n | HR |
| 31 | 40.15 | ghi | S |  | 111 | 4.9 | n | HR |
| 39 | 40.11 | ghi | S |  | 34 | 4.84 | n | HR |
| 4 | 40.11 | ghi | S |  | 27 | 4.68 | n | HR |
| 105 | 40.08 | ghi | S |  | 96 | 3.82 | n | HR |

**Supplementary Table 7.** PVY incidence of different potato germanium resources.

| Resource Number | Incidence rate | Multiple Comparisons |  | Resource Number | Incidence rate | Multiple Comparisons |
| --- | --- | --- | --- | --- | --- | --- |
| 121 | 99.97 | A |  | 37 | 98.61 | A |
| 83 | 99.77 | A |  | 110 | 98.52 | A |
| 138 | 99.67 | A |  | 109 | 98.45 | A |
| 90 | 99.65 | A |  | 46 | 98.45 | A |
| 69 | 99.6 | A |  | 117 | 75.41 | B |
| 93 | 99.6 | A |  | 70 | 75.3 | B |
| 94 | 99.59 | A |  | 4 | 75.11 | B |
| 68 | 99.52 | A |  | 1 | 75.08 | B |
| 54 | 99.51 | A |  | 3 | 75.08 | B |
| 137 | 99.51 | A |  | 71 | 75.07 | B |
| 135 | 99.51 | A |  | 127 | 75.03 | B |
| 106 | 99.49 | A |  | 126 | 74.96 | B |
| 112 | 99.45 | A |  | 116 | 74.28 | B |
| 44 | 99.43 | A |  | 132 | 67.58 | C |
| 113 | 99.39 | A |  | 115 | 67.1 | C |
| 41 | 99.37 | A |  | 19 | 67.08 | C |
| 86 | 99.3 | A |  | 15 | 67.07 | C |
| 134 | 99.29 | A |  | 42 | 67.01 | C |
| 98 | 99.28 | A |  | 100 | 66.88 | C |
| 60 | 99.25 | A |  | 78 | 66.88 | C |
| 123 | 99.25 | A |  | 31 | 66.82 | C |
| 119 | 99.25 | A |  | 73 | 66.81 | C |
| 56 | 99.23 | A |  | 35 | 66.81 | C |
| 125 | 99.22 | A |  | 5 | 66.81 | C |
| 89 | 99.22 | A |  | 39 | 66.78 | C |
| 23 | 99.22 | A |  | 108 | 66.77 | C |
| 53 | 99.19 | A |  | 67 | 66.77 | C |
| 49 | 99.19 | A |  | 129 | 66.76 | C |
| 2 | 99.19 | A |  | 65 | 66.75 | C |
| 29 | 99.19 | A |  | 61 | 66.75 | C |
| 32 | 99.15 | A |  | 59 | 66.75 | C |
| 21 | 99.15 | A |  | 101 | 66.74 | C |
| 75 | 99.14 | A |  | 114 | 66.72 | C |
| 87 | 99.13 | A |  | 128 | 66.71 | C |
| 99 | 99.13 | A |  | 9 | 66.65 | C |
| 85 | 99.12 | A |  | 43 | 66.64 | C |
| 105 | 99.12 | A |  | 25 | 66.62 | C |
| 17 | 99.12 | A |  | 118 | 66.62 | C |
| 76 | 99.12 | A |  | 8 | 66.62 | C |
| 57 | 99.08 | A |  | 24 | 66.61 | C |
| 62 | 99.07 | A |  | 38 | 66.61 | C |
| 102 | 99.06 | A |  | 82 | 66.6 | C |
| 81 | 99.06 | A |  | 45 | 66.55 | C |
| 22 | 99.05 | A |  | 55 | 66.55 | C |
| 95 | 99.03 | A |  | 12 | 66.49 | C |
| 50 | 99.03 | A |  | 79 | 66.46 | C |
| 97 | 99.03 | A |  | 124 | 66.45 | C |
| 92 | 99.02 | A |  | 28 | 66.42 | C |
| 48 | 99.01 | A |  | 51 | 66.28 | C |
| 107 | 99.01 | A |  | 64 | 66.08 | C |
| 18 | 98.99 | A |  | 122 | 50.19 | D |
| 11 | 98.99 | A |  | 40 | 50.04 | D |
| 72 | 98.98 | A |  | 10 | 50.04 | D |
| 14 | 98.95 | A |  | 120 | 49.85 | D |
| 16 | 98.94 | A |  | 133 | 34.32 | E |
| 58 | 98.93 | A |  | 104 | 33.84 | E |
| 77 | 98.93 | A |  | 84 | 33.74 | E |
| 66 | 98.93 | A |  | 103 | 33.58 | E |
| 7 | 98.89 | A |  | 88 | 33.54 | E |
| 26 | 98.88 | A |  | 34 | 33.5 | E |
| 6 | 98.87 | A |  | 136 | 33.48 | E |
| 131 | 98.85 | A |  | 33 | 33.45 | E |
| 47 | 98.85 | A |  | 96 | 33.41 | E |
| 30 | 98.81 | A |  | 13 | 33.4 | E |
| 74 | 98.81 | A |  | 27 | 33.34 | E |
| 63 | 98.79 | A |  | 130 | 33.28 | E |
| 52 | 98.79 | A |  | 20 | 33.22 | E |
| 80 | 98.79 | A |  | 91 | 33.12 | E |
| 36 | 98.79 | A |  | 111 | 32.56 | E |
